# The sGC Activator Runcaciguat Has Kidney Protective Effects and Prevents a Decline of Kidney Function in ZSF1 Rats

**DOI:** 10.3390/ijms241713226

**Published:** 2023-08-25

**Authors:** Jan R. Kraehling, Agnes Benardeau, Tibor Schomber, Laura Popp, Julia Vienenkoetter, Heidrun Ellinger-Ziegelbauer, Mira Pavkovic, Elke Hartmann, Krystyna Siudak, Alexius Freyberger, Ina Hagelschuer, Ilka Mathar, Joerg Hueser, Michael G. Hahn, Volker Geiss, Frank Eitner, Peter Sandner

**Affiliations:** 1 Bayer AG, Research and Early Development, Pharma Research Center, 42096 Wuppertal, Germany; 2 Novo Nordisk A/S, Global Drug Discovery, T1D-Kidney Disease, 2760 Måløv, Denmark; 3 Vincerx Pharma GmbH, 40789 Monheim, Germany; 4 Division of Nephrology and Clinical Immunology, RWTH Aachen University, 52062 Aachen, Germany; 5 Department of Pharmacology, Hannover Medical School, 30625 Hannover, Germany

**Keywords:** sGC activator, chronic kidney disease, cGMP, runcaciguat, ZSF1

## Abstract

Chronic kidney disease (CKD) progression is associated with persisting oxidative stress, which impairs the NO-sGC-cGMP signaling cascade through the formation of oxidized and heme-free apo-sGC that cannot be activated by NO. Runcaciguat (BAY 1101042) is a novel, potent, and selective sGC activator that binds and activates oxidized and heme-free sGC and thereby restores NO-sGC-cGMP signaling under oxidative stress. Therefore, runcaciguat might represent a very effective treatment option for CKD/DKD. The potential kidney-protective effects of runcaciguat were investigated in ZSF1 rats as a model of CKD/DKD, characterized by hypertension, hyperglycemia, obesity, and insulin resistance. ZSF1 rats were treated daily orally for up to 12 weeks with runcaciguat (1, 3, 10 mg/kg/bid) or placebo. The study endpoints were proteinuria, kidney histopathology, plasma, urinary biomarkers of kidney damage, and gene expression profiling to gain information about relevant pathways affected by runcaciguat. Furthermore, oxidative stress was compared in the ZSF1 rat kidney with kidney samples from DKD patients. Within the duration of the 12-week treatment study, kidney function was significantly decreased in obese ZSF1 rats, indicated by a 20-fold increase in proteinuria, compared to lean ZSF1 rats. Runcaciguat dose-dependently and significantly attenuated the development of proteinuria in ZSF1 rats with reduced uPCR at the end of the study by −19%, −54%, and −70% at 1, 3, and 10 mg/kg/bid, respectively, compared to placebo treatment. Additionally, average blood glucose levels measured as HbA1C, triglycerides, and cholesterol were increased by five times, twenty times, and four times, respectively, in obese ZSF1 compared to lean rats. In obese ZSF1 rats, runcaciguat reduced HbA1c levels by −8%, −34%, and −76%, triglycerides by −42%, −55%, and −71%, and cholesterol by −16%, −17%, and −34%, at 1, 3, and 10 mg/kg/bid, respectively, compared to placebo. Concomitantly, runcaciguat also reduced kidney weights, morphological kidney damage, and urinary and plasma biomarkers of kidney damage. Beneficial effects were accompanied by changes in gene expression that indicate reduced fibrosis and inflammation and suggest improved endothelial stabilization. In summary, the sGC activator runcaciguat significantly prevented a decline in kidney function in a DKD rat model that mimics common comorbidities and conditions of oxidative stress of CKD patients. Thus, runcaciguat represents a promising treatment option for CKD patients, which is in line with recent phase 2 clinical study data, where runcaciguat showed promising efficacy in CKD patients (NCT04507061).

## 1. Introduction

The nitric oxide (NO), soluble guanylyl cyclase (sGC), cyclic nucleotide cyclic guanosine 3′, 5′ monophosphate (cGMP) signaling cascade is a pivotal pathway that regulates many cells, tissues, and body functions. Dysregulation of the second messenger cGMP plays a pivotal role in cardiovascular and cardiopulmonary diseases such as chronic heart failure and pulmonary hypertension [1,2]. It has also been shown that cGMP is a prominent regulator of kidney function [3] and could be involved in the regulation of cortical renal blood flow of afferent and efferent arterioles but also medullary perfusion [4]. In addition, cGMP could impact renin secretion but also the tubular transport mechanism [5,6]. Common comorbidities in kidney disease such as hypertension, diabetes, or obesity lead to endothelial dysfunction and impairment of cGMP production, which can cause chronic kidney disease [7]. Therefore, restoring cGMP signaling could become a powerful treatment option for CKD. However, the aforementioned comorbidities are causing a high oxidative stress burden in the kidney, leading to the oxidation of the sGC and finally resulting in heme-free sGC [8]. Oxidized and heme-free sGC can no longer bind NO, which results in a decline in endogenous cGMP production. Thus, PDE5 and PDE9 inhibitors which prevent cGMP hydrolysis by inhibiting the cGMP degrading PDEs are of limited efficacy under these circumstances since they require a sufficient level of endogenous cGMP. More recently, sGC stimulators and activators were discovered which could bind to sGC and trigger cGMP production [1,2], especially sGC activators which bind the oxidized and heme-free sGC (Apo-sGC) and produce cGMP independently of NO [9]. It has been shown that these first generation sGC activators are kidney protective in models in hypertension-induced CKD models [10,11]. However, the previously used first generation sGC activators had to be applied i.v. (cinaciguat) and caused long-lasting hypotension and were therefore not suitable for chronic treatment of CKD patients. More recently, the discovery and optimization of the sGC activator runcaciguat (BAY 1101042) could overcome limitations of previous compounds [12]. Runcaciguat could be applied orally and binds to oxidized and heme-free sGC (Apo-sGC), leading to a concentration-dependent cGMP production in vitro, resulting in blood vessel relaxation ex vivo and blood pressure reduction in vivo [12]. Runcaciguat has already shown dose-dependent kidney protective effects in vivo in preclinical CKD models with different etiologies [13]. To further extend these previous results, we investigated the kidney protective effects of runcaciguat (BAY 1101042) in ZSF1 rats. The ZSF1 rats are characterized by hypertension and hyperglycemia, as well as insulin resistance, but also have a metabolic phenotype with increased blood lipid levels and a metabolic syndrome, and develop a progressive decline of kidney function associated with increased proteinuria [14]. Therefore, ZSF1 rats combine common comorbidities of CKD and DKD patients. A 12-week treatment with runcaciguat could dose-dependently prevent the decline in kidney function and reduced plasma and urinary biomarkers of kidney damage. Interestingly, runcaciguat also decreased blood glucose, triglycerides, and cholesterol levels in ZSF1 rats, suggesting inhibition of the metabolic dysregulation in this disease model. In summary, our data strongly suggest a beneficial effect of runcaciguat in CKD. To investigate that further, a phase 2 clinical study has been performed (NCT04507061) which investigated the effects of runcaciguat in CKD patients.

## 2. Results

The dose-dependent effects of the sGC activator runcaciguat were studied broadly in ZSF1 rats including effects on kidney function, biomarkers for kidney injury, renal histopathology, blood pressure, and gene expression profiles.

### 2.1. Effects of Runcaciguat on Proteinuria in ZSF1 Rats

**Figure 1:**
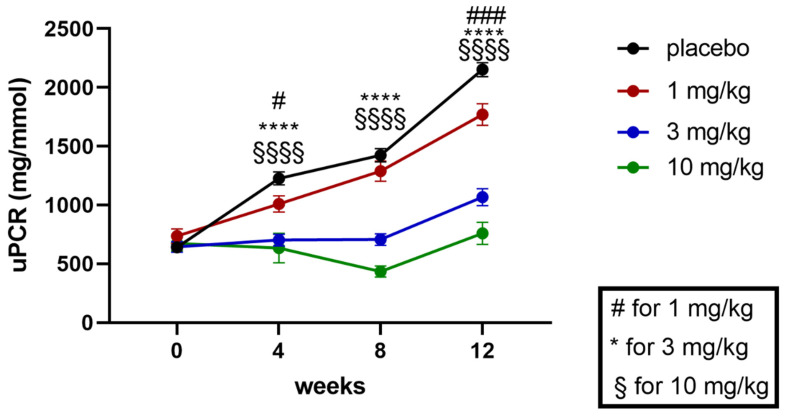
Proteinuria (uPCR) at baseline (at week 0) and during the 12 weeks of treatment study (at weeks 4, 8, and 12 of treatment) in ZSF1 obese rats in [mg/mmol]. Data are mean ± SEM. Significant changes by treatment with runcaciguat were determined by Student’s *t*-test for each time point and dose group with asterisks # for the 1 mg/kg, * the 3 mg/kg), and § for 10 mg/kg dose of runcaciguat with # *p* < 0.05 and ### *p* < 0.001, ****, §§§§ < *p* < 0.0001.

Proteinuria was quantified by analyzing the urinary protein/creatinine ratio at baseline and weeks 4, 8, and 12 (study end) of the treatment. In the 12-week study duration, placebo-treated obese ZSF1 rats were characterized by progressive proteinuria (Figure 1). Runcaciguat treatment was able to dose-dependently attenuate the development of proteinuria already after 4 weeks of treatment in all dose groups. In the 3 and 10 mg/kg treatment arm, the progression of proteinuria was almost completely blunted after 8 weeks of treatment, with a modest increase up to week 12 (Figure 1).

**Figure 2:**
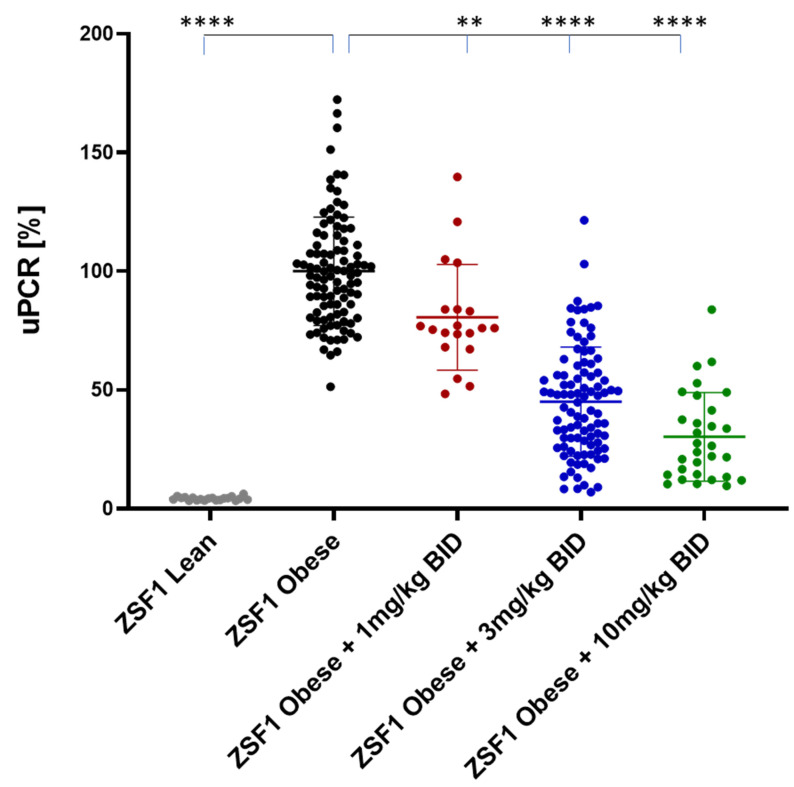
Proteinuria (uPCR) in % of placebo treated ZSF1 obese rats at study end. Lean animals received placebo. Absolute uPCR values at 12 weeks are normalized by the respective mean in the ZSF1 obese placebo group presented on the y-axis. Data are mean ± SE. Significant changes by treatment with runcaciguat versus placebo were determined by one-way ANOVA followed by Tuckey’s multiple comparison test with **/**** for *p* < 0.01/0.0001. A Student’s *t*-test was used to determine a significant change between ZSF1 lean and ZSF1 obese with **/**** for *p* < 0.01/0.0001.

At the study end, proteinuria between ZSF1 lean rats and placebo treated ZSF1 obese rats was increased by 20-fold. ZSF1 obese rats treated with runcaciguat showed a dose-dependent and significant reduction in proteinuria which was −19 ± 4.9%, −54 ± 2.3%, and −70 ± 3.4% at 1, 3, and 10 mg/kg/bid, respectively, compared to placebo (Figure 2).

### 2.2. Effects of Runcaciguat on Urinary Biomarkers of Kidney Injury

**Figure 3:**
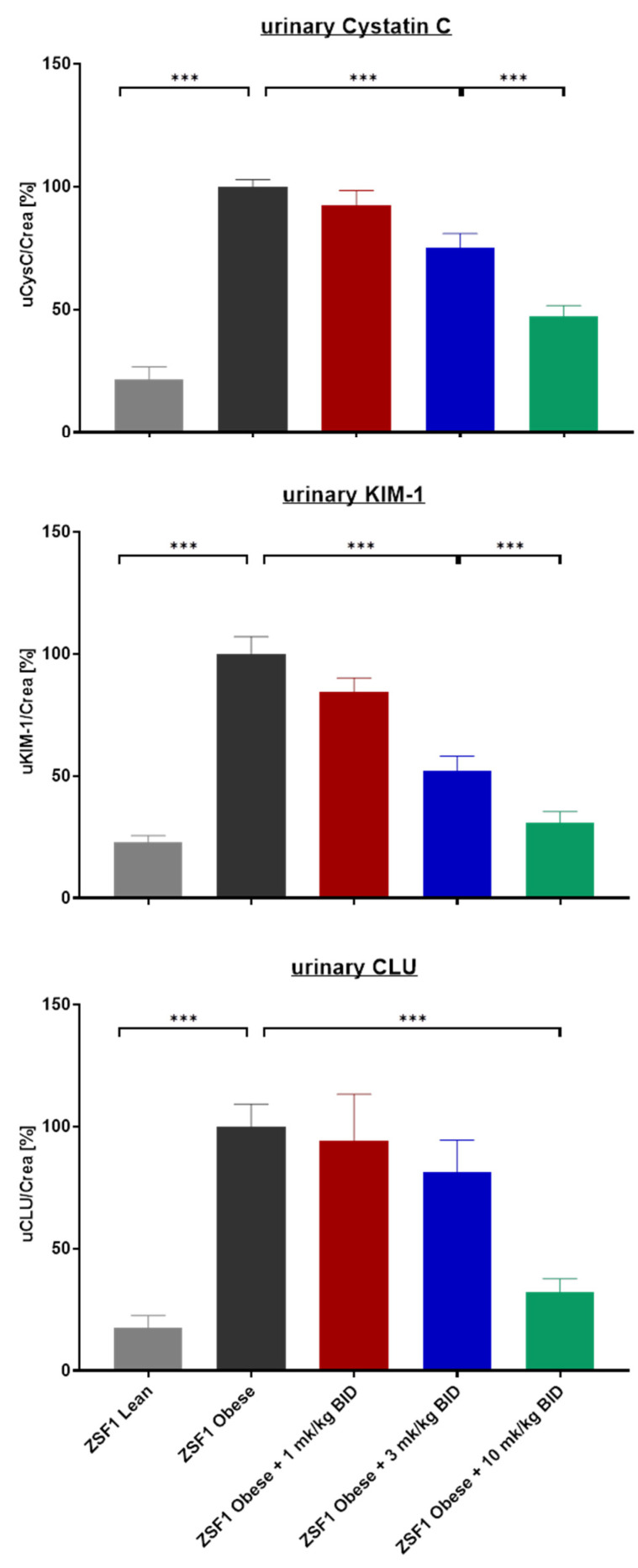
Urinary biomarker Cystatin C, KIM-1, and Clusterin in % of placebo-treated ZSF1 obese rats at study end. Data are mean ± SE. Significant changes by treatment with runcaciguat were determined by one-way ANOVA followed by Tuckey’s multiple comparison test with *** for *p* < 0.001.

In addition to proteinuria, kidney injury biomarkers were quantified in urine. All three biomarkers, cystatin C, KIM-1, and clusterin, were strongly and significantly increased in placebo-treated obese compared to lean ZSF1 rats at the end of the study (Figure 3). Treatment of obese ZSF1 rats with runcaciguat showed a dose-dependent and significant reduction in all biomarkers (Figure 3).

### 2.3. Effects of Runcaciguat on Renal Weights and Histopathology

**Figure 4:**
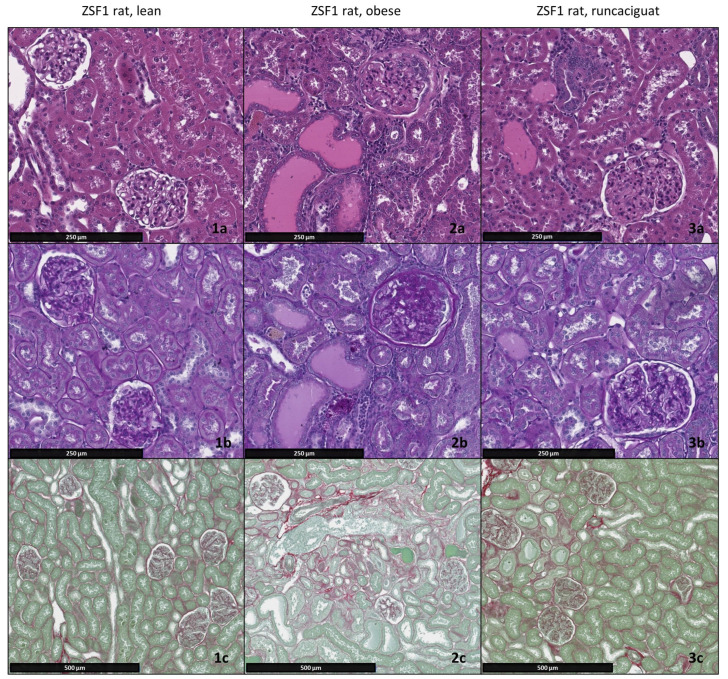
Histopathological changes in renal tissue of 26-week-old ZSF1 lean rats, obese rats, and obese rats treated bidaily with 10 mg/kg runcaciguat for 12 weeks. Shown are representative hematoxylin and eosin (HE)-stained kidney sections, (**a**) periodic acid Schiff (PAS)-stained kidney sections for the detection of glomerulopathy and (**b**) Sirius Red/Fast Green-stained kidney sections for the detection of fibrosis. (**c**) ZSF1 obese rats show moderate (grade 3) protein casts and tubular degeneration, (**2a**) glomerulopathy and (**2b**) multifocally large areas with fibrosis ((**2c**), fibrous tissue in red). Frequently, infiltrates of mononuclear cells were intermingled. In ZSF1 obese rats administered bidaily 10 mg/kg runcaciguat, all parameters (tubular degeneration, glomerulopathy, protein casts, and fibrosis) improved visibly by approximately one severity grade on average (**3a**–**c**) when comparing group means to the untreated obese rats. No histopathological changes were observed in ZSF1 lean control animals (**1a**–**c**).

At the end of the chronic treatment of obese ZSF1 rats, absolute and relative kidney weights of runcaciguat-treated ZSF1 rats showed a significant decrease compared to placebo-treated obese ZSF1 rats (Table 1). In contrast to lean ZSF1 rats which showed no to minimal individual kidney lesions, obese ZSF1 rats displayed clear development of kidney lesions, indicating CKD progression as expected in this animal model. In more detail, the progressive proteinuria in obese ZSF1 rats was accompanied by increasing structural renal damage characterized by moderate glomerulopathy and tubular degeneration with an average score of grade 3. In addition, slight to moderate (grade 2–3) occurrence of protein casts and interstitial fibrosis was found in vehicle-treated obese ZSF1 rats (Figure 4, Table 1). At 10 mg/kg/bid, runcaciguat induced a clear and significant beneficial effect on glomerulopathy, tubular degeneration tubular deposition of protein casts and interstitial fibrosis compared to vehicle-treated obese ZSF1 rats. In addition, 3 mg/kg/bid runcaciguat lead to improvement of the aforementioned parameters (glomerulopathy, tubular degeneration, protein casts, and interstitial fibrosis) although it was not as pronounced as in the higher dose (Table 1). No significant improvement of kidney lesions was observed after chronic treatment with runcaciguat at a dose of 1 mg/kg/bid. However, there was a consistent tendency towards slightly improved values for the parameters evaluated. In summary, runcaciguat 3 and 10 mg/kg were effective dose levels, inducing the most favorable therapeutic effect (Figure 4, Table 1). 

In essence, runcaciguat showed a significant protective effect in ZSF1 rats with a substantial reduction in kidney function decline, preservation of physiological kidney structure, and reduction in kidney damage markers. However, the mode of action of runcaciguat is only partly understood, e.g., runcaciguat could help to maintain kidney perfusion and renal blood flow under oxidative stress conditions by acting on afferent and efferent arterioles [4]. To further complement our understanding of how the beneficial effects of runcaciguat may be meditated we performed additional experiments.

### 2.4. Effects of Runcaciguat on Blood Pressure and RAAS

In preclinical CKD models, hypertension-induced kidney damage represents a very effective driver of CKD progression and decline in kidney function. This is especially seen in CKD models with malignant hypertension but could also play a role in the ZSF1 rats which develop moderate hypertension. Consequently, antihypertensive therapies are highly effective in these preclinical models by simply removing the entire cause or at least a part of the disease driver. Since long-term clinical outcomes are not significantly improved by antihypertensive therapies, it is important to carefully analyze the dose-dependent blood-pressure-lowering effects of runcaciguat in ZSF1 rats and to compare it to the aforementioned, significant kidney protection of runcaciguat in ZSF1 rats.

**Figure 5:**
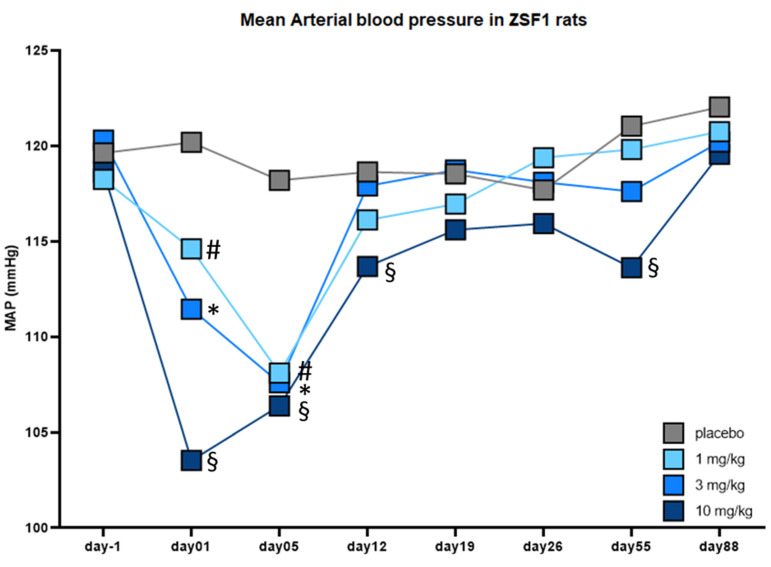
Change of mean arterial blood pressure (MAP) in [mmHg] in conscious obese ZSF1 rats with telemetric implants of runcaciguat treated rats versus placebo. Rats were chronically treated with placebo, 1 mg/kg, 3 mg/kg and 10 mg/kg runcaciguat, and recordings were taken before treatment (day-1) and after initiation of the treatment on day 1, 5, 12, 19, 26, 55, and 88, covering the whole treatment phase of 12 weeks. Data are mean ± SEM. Significant changes by treatment with runcaciguat were determined by *t*-test versus the placebo group #, *, § indicates significance with *p* < 0.05 for 1 mg/kg, 3 mg/kg and 10 mg/kg runcaciguat, respectively.

Therefore, we studied the acute and chronic effects of runcaciguat treatment in ZSF1 rats with telemetric implants. Acute oral dosing of the 1.3 mg/kg and 10 mg/kg runcaciguat led to a dose-dependent and significant decrease in mean arterial blood pressure of −5.6, −8.8 and −16.7 mmHg, respectively, which was still significant on day 5 of treatment in all treatment arms (Figure 5). However, on day 12 of treatment, no significant changes on blood pressure in the 1 and 3 mg/kg runcaciguat treatment group were observed (Figure 5). In addition, the decrease in the 10 mg/kg runcaciguat treatment group was markedly reduced to—4.9 mmHg. On the consecutive days on day 19, 26, 55, and 88, again no significant blood pressure change was detected in the 1 and 3 mg/kg runcaciguat treatment group, but also the blood pressure lowering effect in the 10 mg/kg runcaciguat treatment arm was fading and was no more significant on day 19, 26, and 88 (Figure 5). These data suggest that the significant kidney protective effects as seen in the 12-week treatment study are not primarily mediated by the potential antihypertensive mode of action of runcaciguat, especially not in the 1 and 3 mg/kg dose. 

**Figure 6:**
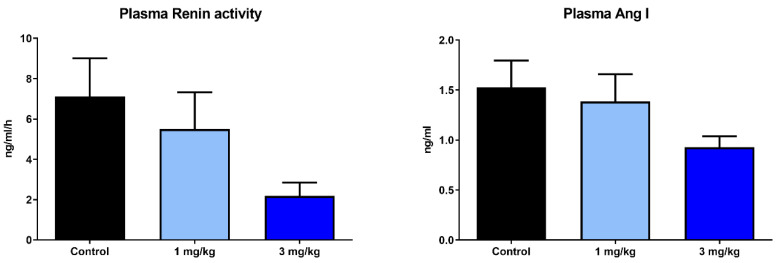
Runcaciguat treatment did not activate the RAAS system, which is in line with the moderate to no blood pressure lowering effects of runcaciguat in obese ZSF1 rats.

Since the activation of the renin–angiotensin–aldosterone system is also a very sensitive parameter for a blood pressure decrease, we also quantified plasma renin activity and plasma angiotensin levels in obese ZSF1 rats treated with 1 and 3 mg/kg runcaciguat after 5 week of treatment (Figure 6). We did not find an increase in RAAS activity with runcaciguat treatment other than a moderate decrease in the 3 mg/kg dose group. This might be interesting for future evaluation more systematically and also in different CKD models.

### 2.5. Effects of Runcaciguat on Diabetic and Metabolic Phenotypes

Since ZSF1 is also characterized by T2D and metabolic syndrome, we were interested if runcaciguat could alter blood glucose and lipids in the obese ZSF1 rat.

#### 2.5.1. Effects on Blood Glucose

**Figure 7:**
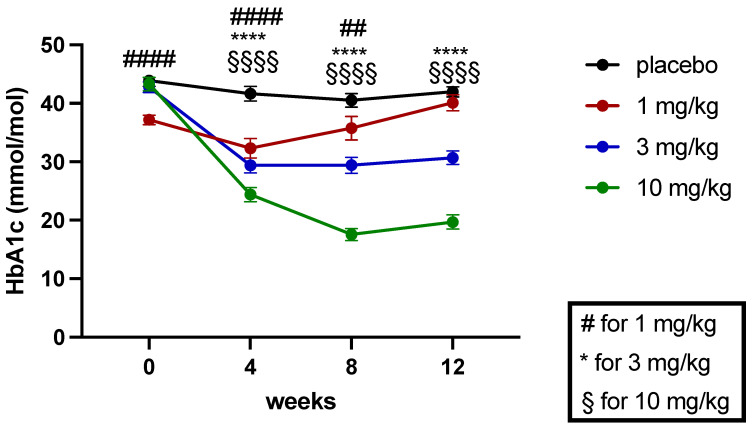
Baseline and progression of HbA1C in mmol/mol in ZSF1 obese rats during the 12-week treatment study. Data are mean ± SEM. Data are mean ± SEM. Significant changes by treatment with runcaciguat were determined by Student’s *t*-test for each time point and dose group with asterisks for *p* < 0.05/0.01/0.001/0.0001. ## *p* < 0.01, ####, ****, $$$$ *p* < 0.0001.

Average blood glucose was quantified by analyzing the Hb (HbA1c) levels and measured at baseline at weeks 4, 8, and 12 (study end) of the treatment. Blood glucose levels remained high in the ZSF1 rats on all time points throughout the study, but HbA1C levels were significantly reduced on week 4 of treatment (Figure 7). During the consecutive weeks of treatment, these effects on HbA1C were fading out in the 1 mg/kg treatment group, remained on the same reduced level in the 3 mg/kg treatment arm, and were further reduced in the 10 mg/kg runcaciguat treatment group (Figure 7).

**Figure 8:**
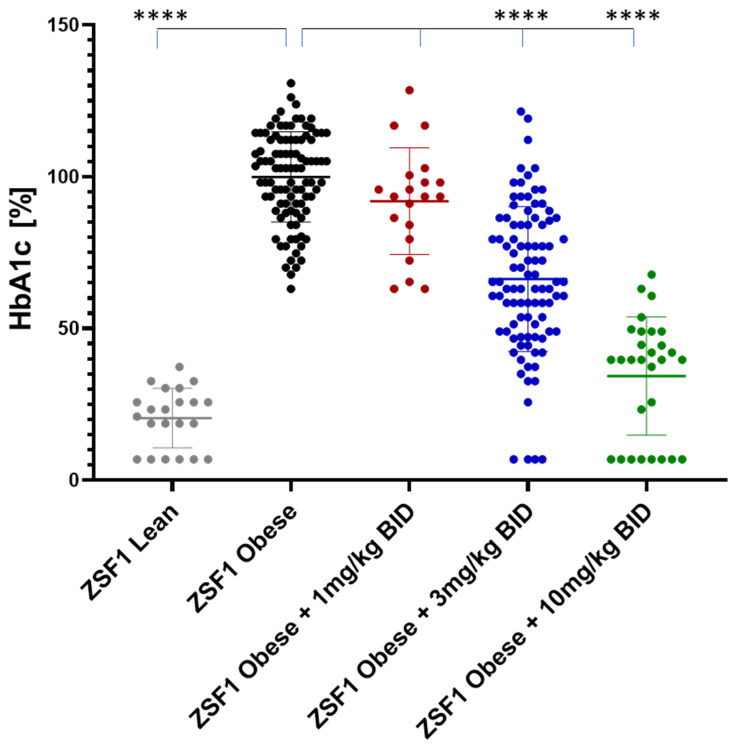
Mean HbA1C levels in % of placebo treated ZSF1 obese rats at study end. Data are mean ± SE. Significant changes by treatment with runcaciguat were determined by one-way ANOVA followed by Tuckey’s multiple comparison test with **** for *p* < 0.0001.

At the study end, blood HbA1c levels were increased around 5-fold between ZSF1 lean and ZSF1 obese rats. Runcaciguat lead to dose-dependent reduction in HbA1c in ZSF1-obese rats by −8 ± 3.8%, −34 ± 2.4% and −76 ± 3.5% at 1, 3 and 10 mg/kg BID, respectively (Figure 8).

#### 2.5.2. Effects on Plasma Triglycerides and Cholesterol

**Figure 9:**
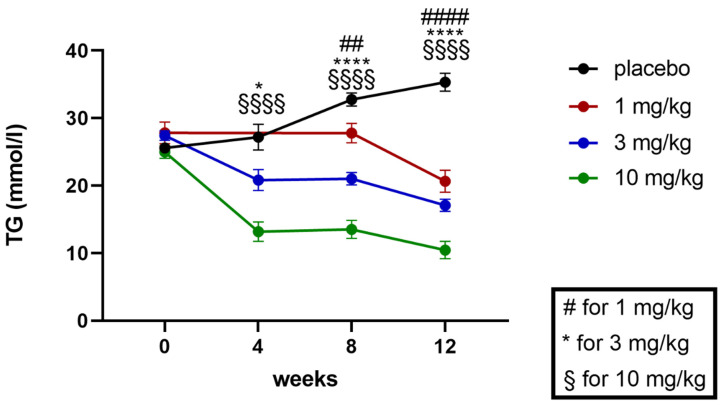
Baseline and progression of TGs in mmol/l in ZSF1 obese rats during the 12-week treatment study. Data are mean ± SEM. Significant changes by treatment with runcaciguat were determined by Student’s *t*-test for each time point and dose group with asterisks for *p* < 0.05/0.01/0.001/0.0001. * *p* < 0.05, ## *p* < 0.01, ####, ****, $$$$ *p* < 0.0001.

**Figure 10:**
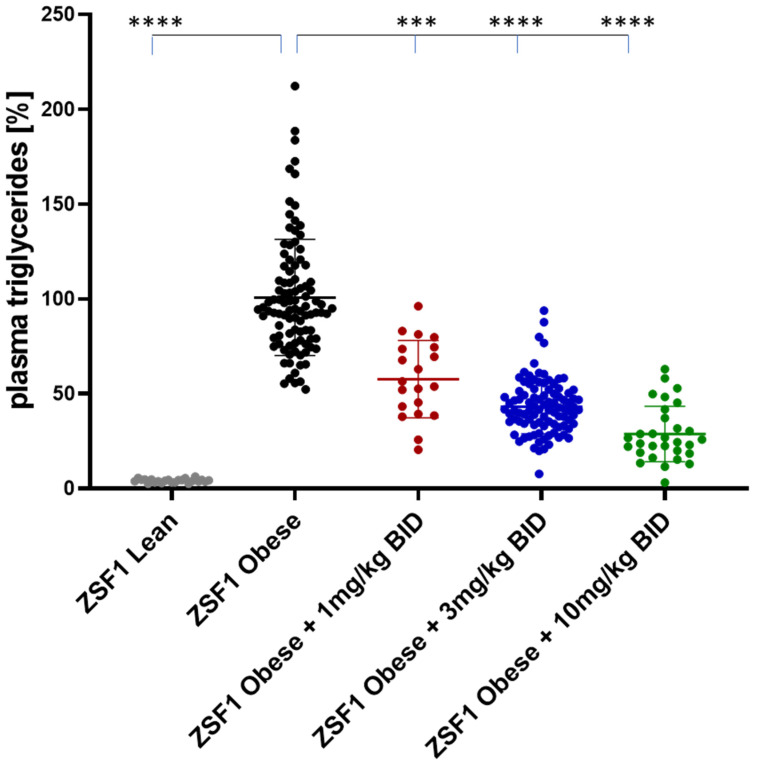
Plasma triglycerides (TG) in % of placebo treated ZSF1 obese rats at study end. Data are mean ± SE. Significant changes by treatment with runcaciguat were determined by one-way ANOVA followed by Tuckey’s multiple comparison test with ***/**** for *p* < 0.001/0.0001.

Plasma triglycerides (TG) were quantified at baseline at weeks 4, 8, and 12 (study end) of the treatment (Figure 9 and Figure 10).

During the course of the study, plasma TG levels were increasing in the placebo treated ZSF1 rats (Figure 9). Treatment with runcaciguat decreased TG levels dose-dependently and significantly in all groups starting at week 4 in the 3 mg/kg and 10 mg/kg runcaciguat treatment group and on week 8 in the 1 mg/kg runcaciguat group (Figure 9).

At the study end, plasma TG levels were increased around 20-fold between ZSF1 lean and ZSF1 obese rats. Runcaciguat lead to dose-dependent reduction in TG levels in ZSF1 obese rats by −42 ± 4.2%, −55 ± 9.9% and −71 ± 5.2% at 1, 3 and 10 mg/kg BID, respectively (Figure 10).

**Figure 11:**
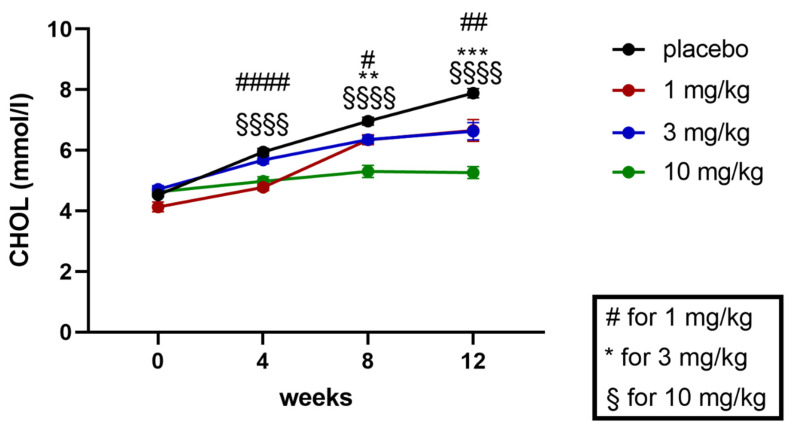
Baseline and progression of cholesterol in mmol/l in ZSF1 obese rats during the 12-week treatment study. Data are mean ± SEM. Significant changes by treatment with runcaciguat were determined by Student’s *t*-test for each time point and dose group with asterisks for *p* < 0.01/0.001. # *p* < 0.05, ##, ** *p* < 0.01, *** *p* < 0.001, ####, $$$$ *p* < 0.0001.

In addition to plasma TGs, plasma cholesterol levels were quantified at baseline at weeks 4, 8, and 12 (study end) of the treatment. Like TGs, cholesterol levels were also increasing during the course of the study in the placebo treated obese ZSF1 rats (Figure 11). Treatment with runcaciguat was able to attenuate cholesterol increase at 1 and 3 mg/kg runcaciguat treatment. Starting at week 4, in the 10 mg/kg runcaciguat treatment arm, the increase in cholesterol levels was completely blunted (Figure 11). 

**Figure 12:**
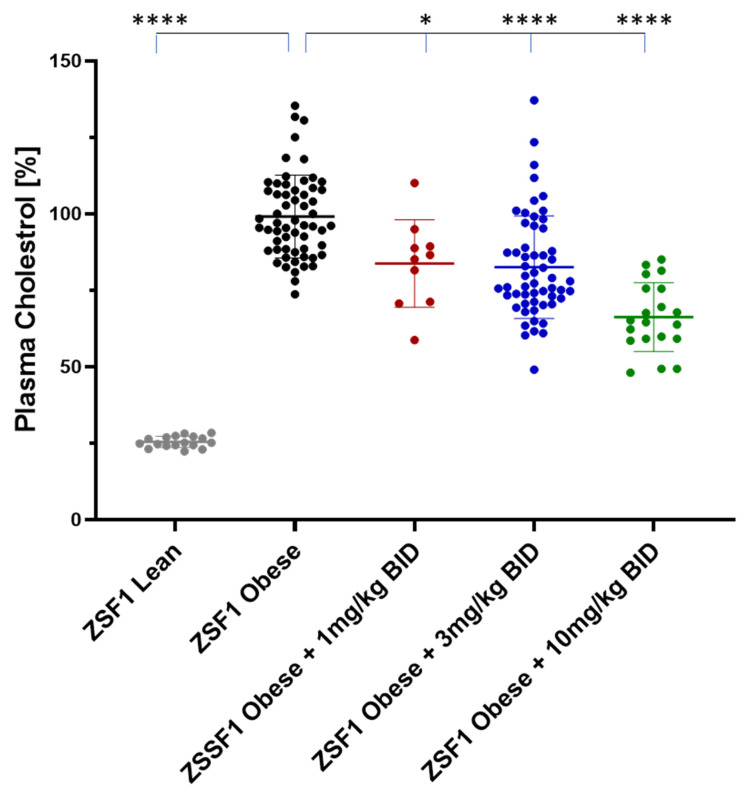
Plasma cholesterol in % of placebo treated ZSF1 obese rats at study end. Data are mean ± SE. Significant changes by treatment with runcaciguat were determined by one-way ANOVA followed by Tuckey’s multiple comparison test with */**** for *p* < 0.05/0.0001.

At the study end, plasma cholesterol levels were increased around 4-fold between ZSF1 lean and ZSF1 obese rats. Runcaciguat lead to dose-dependent reduction in cholesterol levels in ZSF1-obese rats by −16 ± 4.5%, −17 ± 3.0% and −34 ± 2.5 at 1, 3, and 10 mg/kg BID, respectively (Figure 12).

In summary, runcaciguat treatment was able to significantly reduce average blood glucose levels (measured as HbA1C), plasma triglycerides, and cholesterol. These additional impact of runcaciguat on glycemic control and lipids might contribute to the overall efficacy of runcaciguat in CKD, especially with a diabetic and metabolic etiology.

### 2.6. Effects of Runcaciguat on the Regulation of Different Pathways

**Figure 13:**
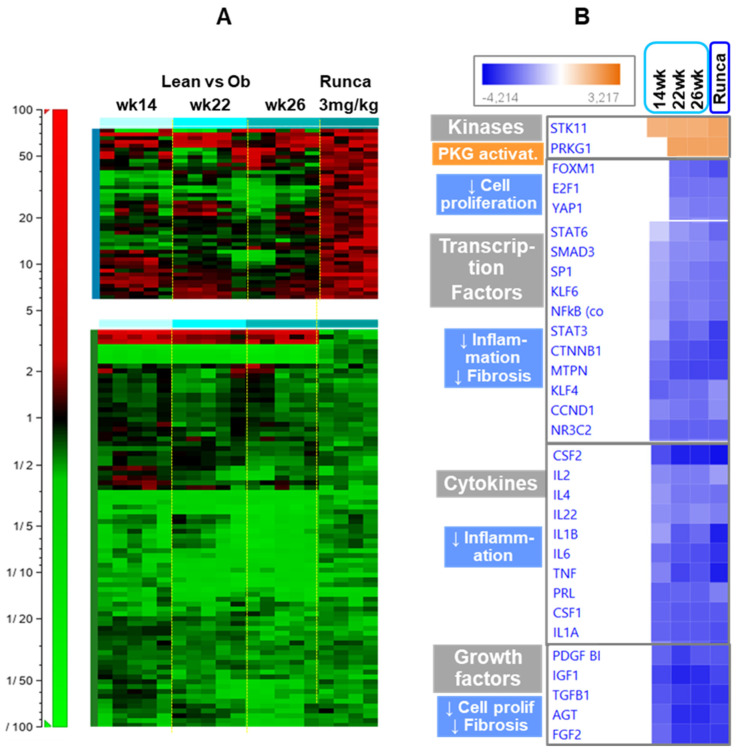
(**A**) Heatmap representing the expression profiles of transcripts significantly affected by 3 mg/kg bid runcaciguat (Runca) in the kidney, and their expression pattern along with disease progression of ZSF1 obese rats. Shown are renal expression profiles of 14-, 22- and 26-week-old ZSF1 lean rats relative to time-matched obese ZSF1 rats, and of—16-week-old obese ZSF1 rats treated for 12 weeks with runcaciguat relative to the matched ZSF1 obese controls. The genes with their corresponding expression ratios are listed in Supplementary Table S3. (**B**) Ingenuity pathway analysis results for genes deregulated by runcaciguat in ZSF1 rat kidney, compared to disease progression in ZSF1 obese rats. Each row represents predicted activation (orange) or inhibition (blue) of kinases, transcription factors, cytokines, or growth factors based on deregulation of their target genes in the dataset shown in Figure 13A (heatmap). Interpretations of these predictions concerning decreased (arrow pointing down) cell proliferation, inflammation, and fibrosis are indicated on the left.

To further elucidate the potential mode for the kidney protective effect of runcaciguat in ZSF1 obese rats, the renal expression profile of genes affected by 3 mg/kg/bid runcaciguat treatment of obese ZSF1 rats between 16 and 27 weeks of age was analyzed with microarray technology and compared to gene expression changes of lean relative to obese ZSF1 rats aged 14, 22, and 26 weeks (Figure 13A heatmap). With the selected deregulation thresholds, 45 and 82 genes were expressed at a higher and a lower level after runcaciguat treatment, respectively. Most of the genes decreased by runcaciguat also show decreased expression in lean vs obese ZSF1 rat kidneys, suggesting that runcaciguat converts the kidney expression profile of obese ZSF1 rats partly to the lean pattern. Most of these genes encode proteins involved in fibrosis, inflammation, and degeneration/regeneration which is supported by ingenuity pathway analysis (IPA) (Figure 13B IPA analysis). 

## 3. Discussion

Despite the control of blood pressure by the use of ACE inhibitors or AT1 receptor blockers and significant progress in the treatment of CKD due to recent approvals of the SGLT2 inhibitor dapagliflozin (Forxiga^TM^) and the non-steroidal MR antagonist finerenone (Kerendia^TM^), progressive decline in kidney function remains an indication with a significant medical need [15,16]. In 2017, the global prevalence of CKD was 9.1% (697.5 million) with more than 160 million patients with DKD ending up with end-stage kidney disease and need for replacement therapies [17]. Therefore, intense research and development efforts are still ongoing to broaden our therapeutic opportunities beyond optimizing blood pressure and glycemic control. In our manuscript, we present preclinical data with the sGC activator runcaciguat [12] which exhibits a completely differentiated mode of action compared to currently available treatment approaches based mainly on RAAS or SGLT2 inhibition. Runcaciguat targets and activates the NO-sGC-cGMP pathway, which is crucial for homeostasis and maintenance of the cardiovascular, cardiopulmonary, and cardiorenal systems, but also in a variety of other diseases and vasculopathy [1,7]. This pathway is known to be impaired in pulmonary hypertension and chronic heart failure, but also in CKD [3,7,18]. Consequently, nitrates, PDE5 inhibitors, or sGC stimulators can improve these diseases, as revealed by the approval of nitrates for angina pectoris, PDE5 inhibitors, and sGC stimulators for pulmonary hypertension and chronic heart failure [19]. However, these treatment options for enhancing the NO-sGC-cGMP pathway face limitations since they can either only augment NO and therefore stimulate native sGC or inhibit cGMP degradation. We could show that CKD is characterized by increased oxidative stress in renal tissues, which could lead to oxidation of sGC, rendering it insensitive to NO. In consequence, cGMP production is at least partially impaired, which also limits the efficacy of PDE5 inhibitors. Therefore, the novel mode of action of runcaciguat, which can activate the oxidized and heme-free form of sGC, could be highly efficacious in CKD. Our data in the ZSF1 rat model for CKD, which is characterized by common comorbidities, namely hypertension, T2D, and obesity leading to a high burden of oxidative stress (Supplementary Figure S1), shows that runcaciguat could substantially attenuate the decline in kidney function associated with significantly less renal damage. Moreover, it partially also corrected for the metabolic imbalance in this model by reducing blood glucose and lipid levels. Importantly, we could observe these beneficial effects of runcaciguat in dosages that do not or do only moderately lower blood pressure. These suggest a blood-pressure-independent mode of action of runcaciguat. Data in ZSF1 rats are consistent with the high efficacy of runcaciguat in other nonclinical studies in a variety of hypertensive, diabetic, and metabolic models of CKD and DKD [4,11]. Our data are also in line with previous reports on the sGC activator BI 703704, which also reduced proteinuria in ZSF1 rats significantly and dose-dependently [20] for this sGC activator, a reduction in proteinuria was also observed in blood pressure neutral doses. According to the runcaciguat results, BI 703704 reduced structural kidney damage. Interestingly, BI 703704 also reduced blood glucose levels in obese ZSF1 rats. Very recently, this was also further confirmed by findings with the sGC activator BI 685509, which decreased significantly—in combination with Enalapril—proteinuria in ZSF1 rats, although in this study, the doses used and the combination treatment had a significant antihypertensive effect [21]. Overall, these data suggest that sGC activators could have a class effect on blood glucose and lipids. The mechanism by which cGMP controls HbA1C, TG, and cholesterol is not fully understood yet and could also be different for glucose and lipids. However, we could show that insulin levels were not influenced by runcaciguat and that we did not observe any effect on glucose tolerance (Supplementary Figure S2). Runcaciguat also decreased fasting glucose levels (Supplementary Table S1), but this was much less pronounced compared to the mean glucose levels measured as HbA1C. It could not be ruled out that food consumption and/or effects on energy expenditure could have an impact on these results. However, the body weight of ZSF1 rats in our studies was not significantly different in the sGC activator treatment groups compared to vehicle control (Supplementary Table S2) and does not suggest an influence of fasting or body weight decrease on the results. 

Interestingly, results with the praliciguat sGC stimulator in ZSF1 rats were also recently reported [22]. The praliciguat sGC stimulator led to a reduction in proteinuria that was more pronounced at the higher dose, although all praliciguat doses used were below the threshold for a significant reduction in blood pressure. These data are in line of a treatment study with an undisclosed sGC stimulator (Compound 1) in ZSF1 rats which also showed a reduction in proteinuria in a chronic treatment study [23]. These are very interesting findings, as sGC stimulators target the native wild-type form of sGC, and their efficacy is limited under oxidative stress. In fact, in a phase 2 clinical study in patients with DKD, praliciguat treatment for 12 weeks did not significantly reduce albuminuria compared to placebo in the primary efficacy analysis [24]. Overall, these studies and discrepancies between non-clinical and clinical results with sGC stimulators show that direct head-to-head comparison of sGC activators and sGC stimulators could be very rewarding and are needed to potentially identify the populations of CKD that benefit the most. These comparisons will also help to better understand the balance of nonoxidized native sGC and the impact of oxidative stress and formation of oxidized and heme-free sGC in CKD and DKD. 

In addition to these beneficial findings with sGC activators and sGC stimulators in obese ZSF1 rats, e.g., inhibition of kidney function decline indicated by significant reduction in proteinuria and decrease in kidney damage biomarkers, it is difficult to predict the molecular mechanism of action of runcaciguat in CKD. An increase in cGMP has previously been shown to have beneficial effects on kidney perfusion and renal blood flow, renin secretion, and tubular function [11,13,20]. Furthermore, inflammation and fibrosis could be reduced [21,25]. Therefore, we also aimed to characterize the potential mode of action in our studies. Histopathology demonstrated a pronounced effect on kidney fibrosis, suggesting an antifibrotic effect of runcaciguat that may contribute to the maintenance of kidney function. Since antifibrotic effects have been seen without reduction in blood pressure, this suggests a direct effect of runcaciguat on fibrotic remodeling. There is literature showing that an increase in cGMP by sGC activators has a pronounced antifibrotic effect on the livers, lungs, hearts, and kidneys, but also on the skin, which [26,27,28] is consistent with our findings in the kidneys of ZSF1 rats.

To investigate the additional mode of action, we carefully analyzed the renal gene expression profile using whole genome microarrays. Runcaciguat decreased the expression of genes encoding proteins involved in fibrosis, inflammation, and degeneration/regeneration in the kidney of obese rats with ZSF1 who also showed lower expression in the kidney of lean rats compared to obese ZSF1 rats, suggesting that runcaciguat converts the kidney expression profile of obese ZSF1 rats at least partly to a lean pattern. Ingenuity pathway analysis (IPA) supported this interpretation by predicting (1) activation of the PRKG1 kinase, which also plays a role in decreasing fibrotic gene expression, indicating activation of the sGC-cGMP-PGK (PRKG1) pathway; (2) inhibition of immune regulation-associated transcription factors (NFkB, STAT3, STAT6) and cytokine responses due to decreased expression of mRNAs encoding immune cell proteins and inflammatory regulators, indicating reduced inflammation; (3) inhibition of transforming growth factor beta (TGFB1) signaling and its associated transcription factors (SMAD3, KLF6), based on decreased expression of mRNAs encoding extracellular matrix (ECM) proteins such collagens and fibronectin, among others, likely correlating with decreased histologically observed fibrosis; and (4) inhibition of transcription factors and regulators playing a role in cell proliferation (E2F1, FOXM1 CCND1) due to decreased expression of mRNAs encoding proteins involved in cell cycle progression, e.g., cyclic spindle components. The latter can be interpreted as the reduction in ongoing regeneration processes, which in the kidney are closely associated with preceding degeneration events. This is in alignment with a very recently published gene expression analysis at single-cell resolution in ZSF1 kidneys after runcaciguat treatment [29]. Specifically, they report the largest numbers of genes returning to a healthy control level from runcaciguat treatment in proximal tubule regions. Thus, we hypothesize, also not investigated by single-cell analysis in the study reported here, that the specific cell types affected include to a large extent proximal tubule, stromal, and mesenchymal cells of the proximal tubule regions. We observed decreased expression of genes also reported by Balzer et al. [29] in this region including *secreted phosphoprotein-1* (*Spp1*), *fibronectin 1* (*Fn1*), and *collagen alpha 1* (*Col1a1*).

Runcaciguat-induced increased expression of genes playing roles in lipid or carbohydrate metabolism (*Mlxipl*, *Tkfc*, *Slc2a5*, *Pklr*, *Fasn*, *Apoc2*, *Acot12*, *Ppara*, *Zbtb16*) suggests increased metabolic turnover. Since not all these genes showed a similar profile in the lean vs. obese comparison, this may represent an adaptation to cope with the diabetic phenotype of the ZSF1 rat model. Interestingly, deregulation of some genes also suggests potential endothelial stabilization, indicated by, for example, increased expression of *Timap* (*TGF-beta-inhibited membrane-associated protein*) which is reported to promote angiogenesis in human glomerular endothelial cells [30], and by decreased expression of *connexin 43* (*Gja1*). Reduced expression of *Gja1* has been reported to improve endothelial structural [31] and increases podocyte health [32]. 

With respect to decreased HbA1c, cholesterol, and TG levels in the blood after runcaciguat treatment, further gene expression analysis in adipose tissue () indicated that the effects may be the result of the interaction of different organs with adipose tissue, the latter potentially playing a prominent role. However, these hypotheses generating data will need to be tested in further specifically designed studies to better understand the mode of action of runcaciguat resulting in a healthier metabolic phenotype.

In essence, given the high efficacy of the sGC activator runcaciguat in ZSF1 rats, as indicated by a substantial reduction in proteinuria that almost blunts the progression of proteinuria in this model, activation of sGC could be a highly effective treatment approach for CKD. Meanwhile, runcaciguat was clinically investigated in a phase 2 clinical study in patients with CKD (CONCORD, NCT04507061) including patients with and without diabetes, but also with and without receiving SGLT2i treatment. The results of this study were very recently presented at the ERA meeting 2023 (ERA-EDTA, Late Clinical Breaker by Ron T. Gansevoort, 16 June 2023) and runcaciguat treatment caused a significant reduction in proteinuria even on top of SGLT2 use. Therefore, sGC activators could present an additional effective treatment approach in CKD with a novel mode of action, which hopefully helps to cover the still high unmet medical need in patients with CKD.

## 4. Material and Methods

### 4.1. Animal Experiments

All experiments and studies were performed according to the guidelines approved by the local animal welfare authorities for the German state of North-Rhine Westphalia (Landesamt für Natur, Umwelt und Verbraucherschutz (LANUV) Nordrhein-Westfalen; N0400a022) and by the institutional animal care and use committee of Bayer AG. All experiments were conducted at the Wuppertal Research Center of Bayer AG. 

### 4.2. Study Protocol

Animals: For the studies, male, obese ZSF1 rats (ZSF1-Lepr^fa^Lepr^cp^/Crl) and male, lean ZSF1 rats (ZSF1-lean) were used. The obese ZSF1 rat is a hybrid rat obtained by crossing a ZDF female and an SHHF male rat. The rats were received from Charles River Laboratories Inc. (251 Ballardvale St, Wilmington, MA, USA). Rats were randomized at the start of the 12-week chronic treatment studies at an age of at least 13 to 14 weeks. This age and treatment duration allows mimicking the CKD phenotypes, including progressive proteinuria and kidney damage. Animals were randomly assigned to treatment groups based on the urinary protein–creatinine ratio. In total, 8 independent studies in ZSF1 rats were conducted, and data were pooled for analysis. The sGC activator runcaciguat (BAY 1101042, [12]) was dissolved in a vehicle consisting of 10% ethanol, 40% Kolliphor^®^ HS15, and 50% water. The runcaciguat solutions were prepared fresh every week, stored at room temperature, and carefully stirred at least 30 min before dosing. Runcaciguat was dosed orally at 1 mg/kg, 3 mg/kg, and 10 mg/kg bidaily and administered by gavage. Placebo controls received the solvent by bidaily gavage. Throughout all studies, a 12-h light/12-h dark cycle was maintained and tap water and a Purina 5008 diet (Sniff Spezialdiäten GmbH, Soest, Germany) were provided ad libitum. For psychological/environmental enrichment, animals were provided with wooden chew blocks (Tapvei Estonia OÜ, Harjumaa, Estonia). Urine collection was performed at baseline and regularly during the studies. Rats were placed in metabolic cages for diuresis for 6–8 h. Prior to necropsy, blood was collected from peripheral veins under deep isoflurane anesthesia. Blood samples were transferred into EDTA-tubes, then processed to plasma that was stored at −20 °C until analyzed. 

### 4.3. Measurement of Urinary Biomarkers

Urine was collected overnight for measurement of standard functional parameters and biomarkers at baseline and at weeks 4, 8, and 12 of the treatment study. Total urinary protein (pyrogallol red-molybdate method) and creatinine according to Jaffe were measured using a Siemens XPT autoanalyzer employing corresponding Siemens test kits. All biomarker assays were performed according to manufacturers’ instructions: Cystatin C: Mouse/Rat Cystatin C Quantikine ELISA, R&Dsystems; KIM-1: Rat TIM-1/KIM-1/HAVCR Quantikine ELISA Kit, R&Dsystems; Clusterin: Rat Clusterin Kit, Mesoscale Discovery. All urinary parameters and biomarkers were normalized to corresponding urinary creatinine values.

### 4.4. Measurements of Plasma Parameters

Blood sampling was performed before the start of the study, at week 4, week 8, and week 12 at the study end. Blood samples were collected by caudal vein puncture or retrobulbar and by aortic bleeding under deep anesthesia at the end of the study, and plasma samples for further measurements were collected in EDTA-coated tubes. All plasma measurements were performed according to manufacturers’ instructions. Glycated hemoglobin (HbA1C) and TG were measured by means of a XPT analyzer, and CHOL was determined using a Cobas c501 autoanalyzer applying corresponding test kits. Renin and angiotensin were measured by RIA (#5309, DRG Instruments GmbH, 35039 Marburg, Germany). 

### 4.5. Microarray Expression Profiling in Kidney

For array-based gene expression analysis kidney samples from the following animals were used: (1) ZSF1 obese rats treated with vehicle or 3 mg/kg/bid runcaciguat between 16 and 27 weeks of age, group size of 4 to 5, and (2) untreated ZSF1 lean and obese rats aged 14, 22, and 26 weeks, group size of 5. Total RNA from ca 70 mg of the kidney was extracted using the RNeasy Mini Kit (Qiagen, Hilden, Germany), according to the manufacturer’s instructions. RNA quantity was determined with a Nanodrop^®^ 1000 Spectrophotometer, and quality was assessed with Bioanalyzer^®^ RNA 6000 Nano Kits (Agilent, Santa Clara, CA, USA). Biotin-labeled copy-RNA prepared from 300 ng total kidney RNA was processed and hybridized on rat Clariom^TM^ D assay arrays representing all known rat genes (Affymetrix by Thermo Fisher Scientific Inc., Waltham, MA 02451, USA) according to the manufacturer’s instructions and scanned using the Affymetrix GeneChip Scanner 3000 (Thermo Fisher Scientific Inc., Waltham, MA 02451, USA). 

Using Genedata^®^ Analyst, genes significantly deregulated by runcaciguat compared to vehicle-treated ZSF1 obese rats were identified with a *t*-test (*p*-value ≤ 0.05) combined with at least 1.6-fold deregulation. After filtering out 9 genes with an intensity < 20 in half of the samples, a final list of 127 genes was obtained. The expression profiles of these genes in runcaciguat vs. vehicle-treated rats were then visualized alongside lean vs. obese ZSF1 rats in a heatmap, and subjected to ingenuity pathway analysis (IPA, Ingenuity^®^ Systems, www.ingenuity.com, assessed on 27 July 2020). With the so-called upstream analysis of IPA, the in- or decreased activity of chemicals, cytokines, enzymes, or transcription factors was predicted based on the direction of deregulation of genes described in this context.

### 4.6. Necropsy and Histopathology

At the end of each study, animals were kept in deep anesthesia (isoflurane, 5–10%) and sacrificed by exsanguination via cut of axillary vessels. Kidneys were harvested, weighed, rinsed, and then fixed for histological evaluation or immediately frozen for analysis of expression of kidney damage marker genes. Kidney samples for histology were fixed in Davidson’s solution and embedded in paraffin. Paraffin sections (approx. 5 µm) were prepared and stained with hematoxylin and eosin (HE), periodic acid–Schiff (PAS; detection of glomerulopathy and protein casts) and Sirius Red/Fast Green (SR/FG; detection of interstitial fibrosis). The slides were analyzed by light microscopy using a semiquantitative scoring, ranging from grade 1 to 5 (grade 1, minimal/very few, the lesion is of small quantity and extension and barely exceeds control limits; grade 2, slight/few/small, the lesion is easily identifiable but of limited severity and extension; grade 3, moderate, the lesion is prominent and expanded to a greater degree but there is significant potential for increased severity; grade 4, marked/many, the lesion is prominent but not yet as complete as possible; grade 5, massive, the degree of change is as complete as possible, i.e., occupies the majority of the organ) [33]. The grading was applied for each of the predominant kidney lesions such as glomerulopathy, tubular degeneration, protein casts, and interstitial fibrosis by a certified pathologist.

Glomerulopathy was characterized primarily by degenerative changes affecting the glomerulus. Features include e.g., focal to segmental or global lesions such as hypercellularity and enlargement of the glomerulus (glomerulonephritis), shrinkage of the glomerular tuft, replacement of the mesangium by fibrosis (glomerulosclerosis), synechiae formation, basement membrane thickening, or crescent formation. The term tubular degeneration was chosen to subsume a variety of morphologic features including cellular swelling, pale and/or basophilic staining, peritubular basement membrane thickening, and tubular dilation. Protein casts consisted of homogeneous PAS-positive content filling the tubular lumen. Interstitial fibrosis was diagnosed based on accumulation of fibrous collagen (red in the SR/FG stain) with an increase in interstitial cells.

### 4.7. Blood Pressure Measurements

Hemodynamics were recorded either via tail-cuff or in ZSF1 rats with telemetric implants in a separate cohort of animals. Blood pressure and heart rate were monitored in freely moving conscious animals by radio telemetry as described previously in [34]. Briefly, the telemetric system (DSI Data Science International, St. Paul, MI, USA) was used, and transmitters (TA11PA-C40) were implanted in ZSF1 rats, according to the DSI guidelines, and the tip of the telemetric catheter was placed caudal to the renal arteries and secured by tissue adhesive. After recovery, rats were then housed individually and the individual radiotelemetric signals were registered by RA1010 receiver plates under each cage and stored and processed by Dataquest A.R.T 4.0 for Windows which converts telemetric pressure signals to mmHg. Data collection was started 2 h before drug administration and finished after completion of 24 h cycles for the acute experiments, whereas for chronic monitoring, data were collected over 10 days with the first day without drug treatment and days 9 and 10 as a washout.

### 4.8. Statistical Analysis

All analyses were performed using GraphPad Prism software v8 (GraphPad Software, San Diego, CA, USA). To identify outliers, the ROUT test with Q = 1% was used. To identify statistical differences between groups, a one-way ANOVA was first performed, and if significant this was followed by post-hoc Tuckey’s multiple comparisons test; *p* < 0.05 was considered as significant.

## Figures and Tables

**Table 1:** Kidney hypertrophy and histopathological grading in obese ZSF1 rats treated bid with either placebo or 1 mg/kg, 3 mg/kg and 10 mg/kg runcaciguat. Data are mean ± SE., Significant changes by treatment with runcaciguat versus placebo were determined by one-way ANOVA followed by Tuckey’s multiple comparison test with * for *p* < 0.05.

ZSF1 Obese Rats Treatment Groups	Kidney Weight/Body Weight [g/kg]	Glomerulopathy [Grade]	Tubular Degeneration [Grade]	Protein Casts [Grade]	Interstital Fibrosis [Grade]
ZSF1 obese + placebo	7.17 ± 0.34	3.28 ± 0.57	2.94 ± 0.31	2.63 ± 0.34	2.57 ± 0.31
ZSF1 obese + 1 mg/kg BID	6.95 ± 0.50	3.1 ± 0.41	2.85 ±0.01	2.25 ± 0.73	2.4 ± 0.6
ZSF1 obese + 3 mg/kg BID	5.95 ± 0.33 *	2.09 ± 0.68	2.10 ± 0.63	1.69 ± 0.68	1.37 ± 0.67
ZSF1 obese + 10 mg/kg BID	5.45 ± 0.16 *	1.55 ± 0.92	1.15 ± 0.49	1.20 ± 0.14	0.9 ± 0.1

## Data Availability

All raw data and material is available and documented on file.

## Supplementary Materials

The following supporting information can be downloaded at: https://www.mdpi.com/article/10.3390/ijms241713226/s1.

## Abbreviations

ANOVA, analysis of variance; cGMP, cyclic guanosine monophosphate; CKD, chronic kidney disease; bid, bidaily; BW, body weight; CTEPH, chronic thromboembolic hypertension; CreaCl, creatinine clearance; Cyst-C, cystatin C; DKD, diabetic kidney disease; ESRD, end-stage renal disease; g, gram; GRF, glomerular filtration rate; HbA1C, Hemoglobin A1c; HF, heart failure, H-FABP, heart-type fatty acid binding protein; HFrEF, heart failure with reduced ejection fraction, HFpEF heart failure with preserved ejection fraction; HW, heart weight; KIM-1, kidney injury molecule 1; kg, kilogram; KW, kidney weight; L-NAME, Nω-nitro-L-arginine methyl ester; LVR, left ventricle weight; mg, milligram; ml, milliliter; mmHg, millimeter mercury; NGAL, neutrophil gelatinase-associated lipocalin; NO, nitric oxide; OPN, osteopontin; PAH, pulmonary arterial hypertension; PDE, phosphodiesterase; po, per os/oral; ppm, parts per million; qd, once daily; RAAS, renin angiotensin aldosterone system; RenTG, renin transgenic; RVW, right ventricle weight; SHHF, spontaneously hypertensive heart failure rats; SD, Sprague Dawley rats; SEM, standard error of the mean; sGC, soluble guanylyl cyclase; TG, triglycerides, CHOL, cholesterol; T2D, type two diabetes; uPCR, urinary protein-creatinine ratio; ZDF, Zucker-diabetic fatty rats; ZSF1, cross breed of ZDF and SHHF rats.
