# Supplementary material for: The sGC Activator Runcaciguat Has Kidney Protective Effects and Prevents a Decline of Kidney Function in ZSF1 Rats
Source: Int J Mol Sci. 2023 Aug 25;24(17):13226. doi: 10.3390/ijms241713226 (PMC10488129; doi:10.3390/ijms241713226)
Supplement: Supplementary file 1 [file ijms-24-13226-s001.zip › ijms-2532868-supplementary.pdf]

Supplementary information:

Suppl. Figure S1

Staining of oxidative stress by 8OH DG in kidney biopsies from ZSF1 rats

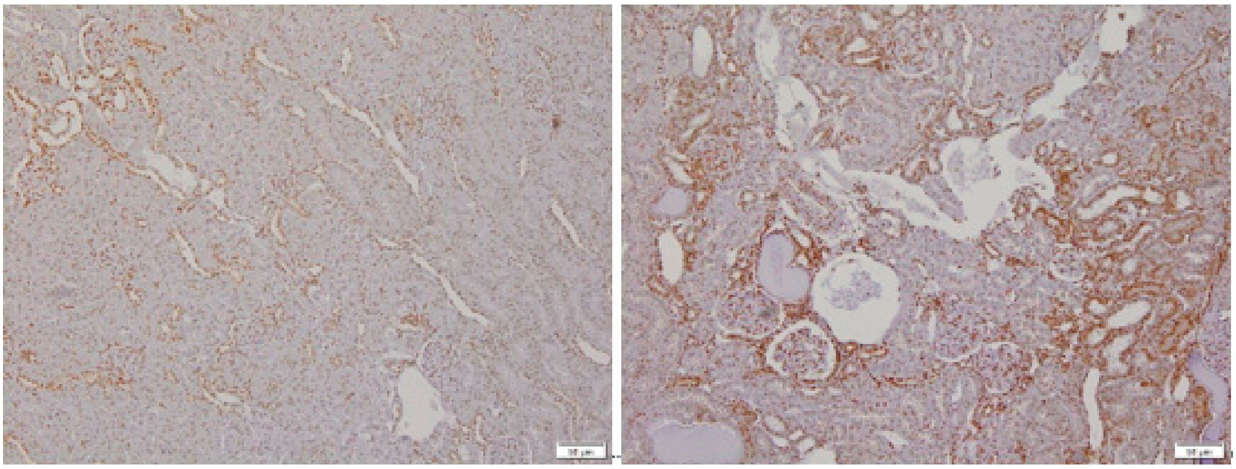

Immuno-staining for 8-OH-dG (8 hydroxy-deoxy-guanosine) performed on kidney biopsies of or male lean control rat (left) or male obese ZSF1 rat (right) rat at the age of 25W. 8-OH-dG stands as marker of oxidative stress damage and repair.

Suppl. Figure S2

Effects of runcaciguat on oral glucose tolerance test (oGTT) in obese ZSF1 rats

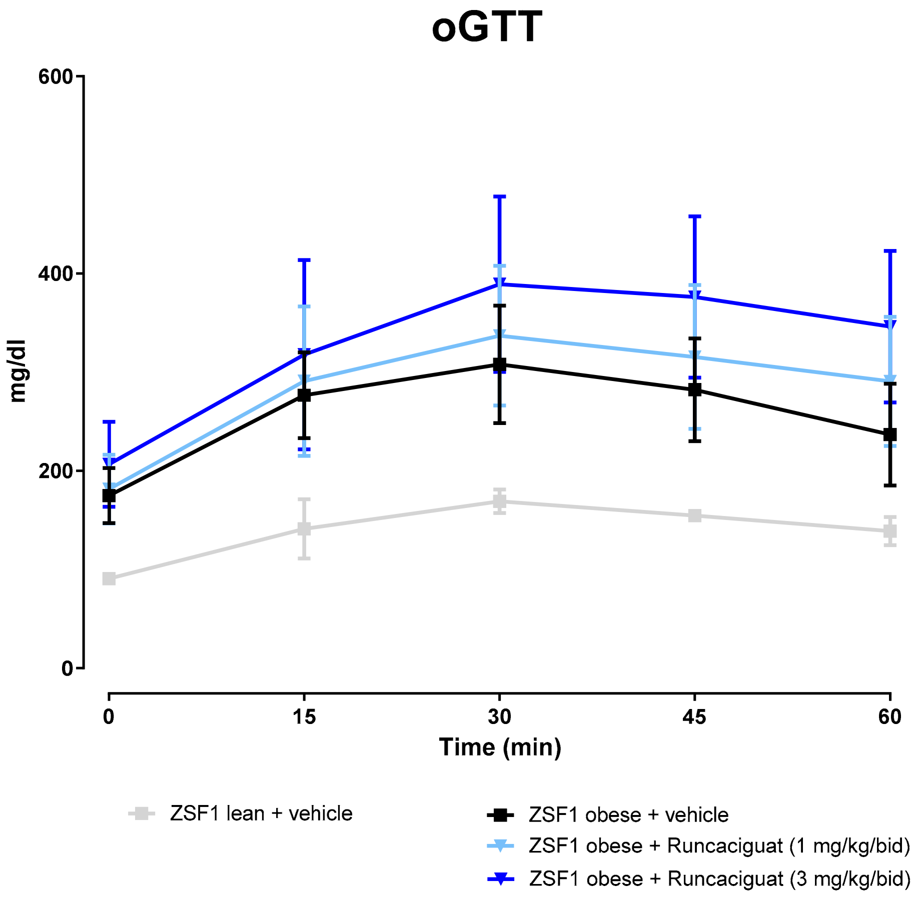

Blood glucose levels in conscious ZSF1 lean and ZSF1 obese rats in [mg/dl] after oral glucose bolus with 1 g/kg at 0 min at 0, 15, 30, 45 and 60 minutes after glucose challenge. Rats were chronically treated with vehicle (placebo), 1 mg/kg and 3 mg/kg runcaciguat for 12 weeks. Data are mean ± SEM with n=6 in ZSF1 lean and n=10 in ZSF1 obese in each group. No significant changes within the ZSF1 obese groups could be detected.

Suppl. Table S1:

Effects of runcaciguat on fasting blood glucose levels in obese ZSF1 rats

Treatment	Vehicle	Runcaciguat 3 mg/kg bid	Runcaciguat 10 mg/kg bid
Fasted glucose [mmol/l]	19.3	14.22**	12.91****
SEM	0.70	1.06	1.51

Fasting blood glucose levels in conscious ZSF1 obese rats in [mmol/l], treated with either runcaciguat or vehicle. Rats were chronically treated with vehicle (placebo), 3 mg/kg and 10 mg/kg runcaciguat for 12 weeks. Data are mean ± SEM with n=10 in each group. Significant changes by treatment with runcaciguat were determined by one-way ANOVA followed by Tuckey's multiple comparison test with */**/***/****/ for P < 0.05/0.01/0.001/0.0001.

Suppl. Table S2

Effects of runcaciguat on body weight development in ZSF1 rats

Treatment	Vehicle	Runcaciguat 1 mg/kg/ bid	Runcaciguat 3 mg/kg bid	Runcaciguat 10 mg/kg bid
Body weight [g]	619	619	641	619
SEM	14	15	16	9

Change of body weight in conscious obese ZSF1 rats of runcaciguat treated rats versus vehicle. Rats were chronically treated with either vehicle (placebo), 1 mg/kg, 3 mg/kg and 10 mg/kg runcaciguat for 12 weeks. Data are mean ± SEM. No significant changes by treatment with runcaciguat were determined.
